# Which clinical factors are predictive of outcome in primary spontaneous pneumothorax management?

**DOI:** 10.1136/bmjresp-2024-003089

**Published:** 2025-06-19

**Authors:** Mohammed Zain Raza, Beenish Iqbal, Anand Sundaralingam, Dinesh Addala, Alguili Elsheikh, Rob Hallifax, The RAMPP Trial collaborators

**Affiliations:** 1 Oxford Centre for Respiratory Medicine, Oxford University Hospitals NHS Trust, Oxford, UK; 2 St Peter’s College, University of Oxford, Oxford, UK; 3 Oxford Respiratory Trials Unit, Oxford University, Oxford, UK; 4 NIHR Oxford Biomedical Research Centre, University of Oxford, Oxford, UK

**Keywords:** Pleural Disease

## Abstract

**Background:**

Primary spontaneous pneumothorax (PSP) occurs when air leaks into the pleural space in patients without known underlying lung disease, causing pain and breathlessness. Optimal management of PSP is not defined and we are unable to predict who will fail medical treatment (ongoing pneumothorax with prolonged air leak). We hypothesised that patients with longer symptom duration and higher symptom scores would be more likely to fail treatment.

**Methods:**

Prospectively collected data from the Randomised Ambulatory Management of Primary Pneumothorax randomised controlled trial of ambulatory management were used to determine which clinical factors are associated with treatment failure including symptom scores, time from symptom onset to presentation, treatment allocation, vital signs, history of prior pneumothorax and size of initial pneumothorax.

**Results:**

Overall, 63/236 patients (26.7%) failed treatment. On average, symptoms started 1 day before admission. Multivariable analysis found that patients who presented at least 1 day after symptoms began had a lower risk of treatment failure than those presenting on the day symptoms began (ORs 0.39 (0.18 to 0.81)).

**Conclusion:**

Further work is required to determine psychological drivers of PSP presentation and risks of prolonged air leak.

**WHAT IS ALREADY KNOWN ON THIS TOPIC**

- Primary spontaneous pneumothorax (PSP) commonly occurs in otherwise fit young patients but around one in four will fail standard treatment and require surgical referral for ongoing air leak. There are currently no good predictors of who will fail treatment.

**WHAT THIS STUDY ADDS**

- We retrospectively analysed robustly collected data from a randomised controlled trial of PSP management. We found that of the clinically relevant parameters, the timing of initial presentation to symptom onset was associated with treatment failure (rather than severity of symptoms or size of pneumothorax).

**HOW THIS STUDY MIGHT AFFECT RESEARCH, PRACTICE OR POLICY**

- Clinicians should be aware that patients presenting on the same day as their symptoms began may have an increased risk of treatment failure. Further research is required to investigate and validate these findings.

Spontaneous pneumothorax is a common pathology with an incidence of 17–24 and 1–6 per 100 000 population per annum for men and women, respectively.^1^ Primary spontaneous pneumothorax (PSP) conventionally refers to patients with no underlying lung disease.^2 3^

The optimal initial treatment regime for PSP is not yet defined.^4^ Options include conservative management, aspiration or insertion of a small-bore chest drain. International guidelines and expert consensus statements vary.^2 3 5^ Recently updated British Thoracic Society (BTS) guidelines suggest treatment should be offered to patients with symptomatic pneumothorax.^3^ Previous guidelines suggested aspiration as initial treatment, followed by chest tube insertion, if unsuccessful.^2^ If admitted, then the average hospital stay is 4–5 days.^6^ Patients with ongoing pneumothorax (ongoing air leak and/or the lung not fully re-expanded on chest radiograph (CXR)) by days 4–5 have ‘failed’ medical treatment and should be referred for surgical management.^2 3^

Currently, there are no validated predictive models for treatment failure in patients with PSP. No prior studies have investigated the relationship between patient symptoms and outcomes.

We hypothesised that patients with longer symptom duration and higher symptom scores would be more likely to fail treatment.

## Methods

Data was collected on the 236 patients enrolled in the Randomised Ambulatory Management of Primary Pneumothorax (RAMPP) study: a randomised controlled trial (RCT).^6^ Standard care was defined by the 2010 BTS Pleural guidelines which recommended initial aspiration but, at the discretion of the treating physician, a chest drain could be inserted.^2^

Treatment failure was defined as ongoing air leak and/or the lung not fully re-expanded on CXR by day 4, at which point the patient was referred for surgery. Large pneumothorax was defined as ≥4 cm (measured at the level of the hilum on baseline CXR) by the investigators at randomisation. Variables included in the analysis were chosen for clinical likelihood of impacting treatment failure based on previous literature and expert opinion: duration of symptoms prior to presentation to hospital, severity of pain and breathlessness symptoms (as measured on a 100 mm Visual Analogue Scale (VAS)^7^), initial clinical observations at admission (heart rate, respiratory rate and systolic blood pressure), previous personal history of pneumothorax, smoking status and size of pneumothorax (large or not).^6^

Statistical analysis was assessed using the χ^2^ test (for discrete binary outcomes) and t-test or Mann-Whitney U test (for continuous outcomes). Multivariate analysis was conducted by logistic regression. Statistical significance was set at 0.05. Statistical analysis was undertaken using Stata (V.14.2).

### Patient and public involvement

This analysis was conducted as a post hoc analysis of a previously conducted randomised controlled trial. As such, patient and public involvement was not involved in this study design or conduct.

## Results

Table 1 shows the demographics of the 236 patients. The majority of patients (193) were male (82%). The mean age at recruitment was 30 years (SD 8), 58 (25%) had a history of previous pneumothorax and 20 (8%) had a family history (first-degree or second-degree relative) of pneumothorax. 161 (68%) patients were current or former tobacco smokers, with a median pack-year history of 8 (IQR 5–12), and 114 (48%) were current or former marijuana smokers. 96 patients (40.7%) presented on the same day their symptoms started (‘Same-day’).

**Table 1:** Patient demographics

Demographic	N/236 (%) or mean (SD)
Gender	
Male	193 (81.8%)
Age (mean, SD)	30 (8)
Tobacco smoking	
Current	117 (49.6%)
Ex-smoker	44 (18.6%)
Never smoker	72 (30.5%)
Unknown	3 (0.6%)
Marijuana smoking	
Current	66 (28.0%)
Ex-smoker	48 (20.3%)
Never smoker	112 (47.5%)
Unknown	10 (4.2%)
Ethnicity	
White	213 (90.3%)
Other	23 (9.7%)
Pneumothorax side	
Left	131 (55.5%)
Size of pneumothorax	
Large (≥4 cm)	138 (58.4%)
Moderate (<4 cm)	98 (41.5%)
Previous pneumothorax	
Yes	58 (24.6%)
No	177 (75.0%)
Unknown	1 (0.4%)
Family history of pneumothorax	
Yes	20 (8.5%)
No	200 (84.7%)
Unknown	16 (6.8%)

### Symptom scores

**Figure 1:**
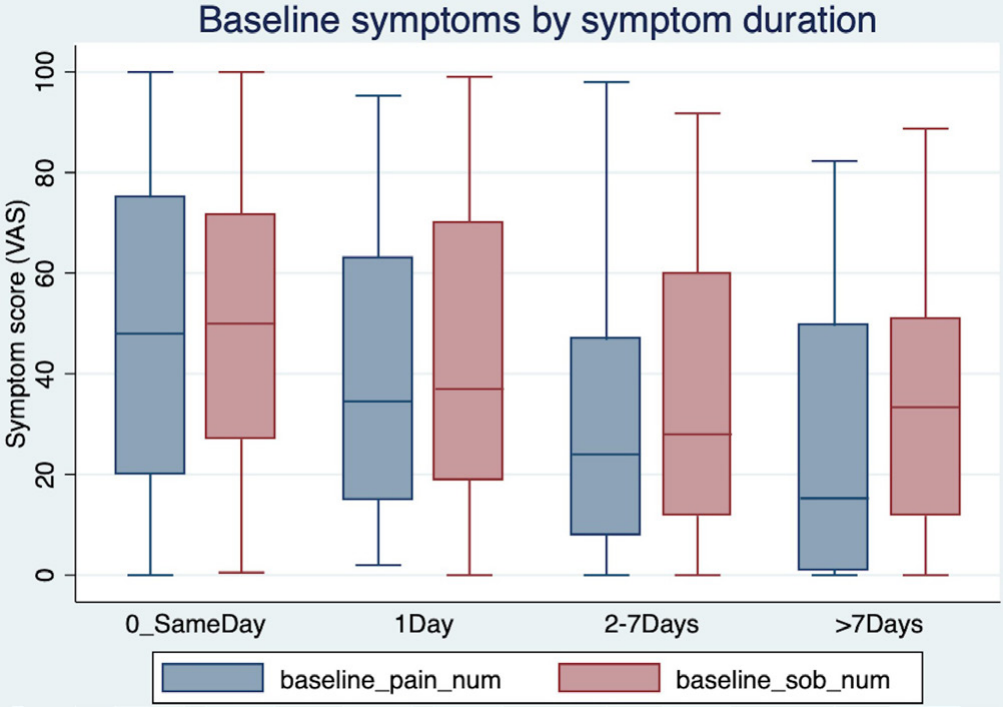
Symptom scores (Visual Analogue Scale pain and breathlessness) at baseline by duration of symptoms prior to admission.

Baseline symptoms scores were available for 215/236 (91.1%). Patients had a median breathlessness score of 42.8 (IQR 19–66.5) and pain score of 38.8 (13.5–65) at baseline. The median duration of symptoms prior to admission to hospital was 1 day (0–3). Same-day admission patients had significantly higher pain and breathlessness scores: median 47.5 (18–75) and 49.5 (27–72), compared with those whose symptoms started at least 1 day prior: 28 (9–51) and 33 (12–63), respectively (p 0.004 and 0.010, Mann-Whitney). Figure 1 shows that the median symptom scores decreased with longer symptom duration: patients whose symptoms started >7 days prior to admission had the lowest symptom scores.

### Vital signs

No patients were in respiratory distress or in cardiovascular shock on admission (patients with signs of tension were excluded from enrolment in the RAMPP study). The mean (SD) baseline respiratory rate, heart rate and systolic blood pressure at enrolment were 19.3 breaths per minute (4.3), 82.6 beats per minute (15.7) and 128 mm Hg (15.6), respectively. The oxygen saturation (on room air) was 96.5% (2.3).

### Treatment failure

**Figure 2:**
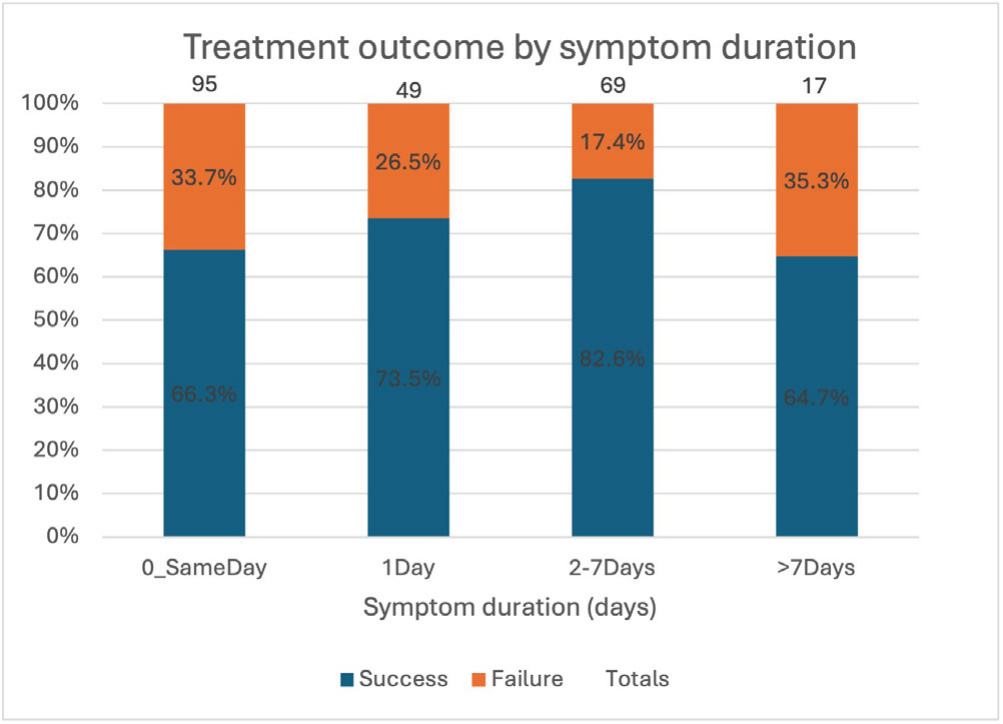
Treatment outcome (failure/success) by symptom duration group.

Data on treatment failure were available for 231 (97.9%) patients. 63 of 231 (27.3%) patients failed medical treatment (ie, had ongoing pneumothorax and at day 4) and so were referred for surgery. Figure 2 shows treatment outcome by symptom duration. Patients whose symptoms started at least 1 day prior to admission had a lower rate of failure (23.0%, 31/136) compared with same-day patients (33.7%, 32/95) (figure 2). 6 of the 17 patients (33.7%) with symptoms for >7 days prior to admission failed treatment, but this was not statistically significant. Patients who failed treatment had a lower heart rate on admission (78.4 beats per minute, SD 15.5) compared with those who spontaneously resolved (84.1 beats per minute, SD 15.6).

### Factors affecting treatment failure—multivariate analysis

Table 2 shows odds of treatment failure by clinical factors (univariable and multivariate analyses). There was no significant difference in the risk of treatment failure by gender, intervention arm, previous pneumothorax, size of pneumothorax, smoking status, duration of symptoms, baseline pain or breathlessness scores, respiratory rate or systolic blood pressure on admission. Patients whose symptoms started at least 1 day prior to admission and patients who failed treatment had a lower heart rate on admission and had OR for treatment failure of 0.6 (95% CI 0.3 to 1.1) and 0.78 (0.63 to 0.96) (per 10 beats per minute), respectively. These two factors remained significant on multivariable analysis with OR 0.4 (0.2 to 0.8, p 0.012) for duration of symptoms >1 day and OR 0.79 (0.63 to 0.997) (per 10 beats per minute) (p 0.047) for heart rate.

**Table 2:** Clinical factors and odds of treatment failure (univariable and multivariate analyses)

		Univariable analysis		Multivariate analysis	
Variables (categorical)	Treatment failure n/N (%)	Odds ratio (95% CI)	P value	Odds ratio (95% CI)	P value
Gender					
Male	48/189 (25.4%)				
Female	15/42 (35.7%)	1.63 (0.8 to 3.32)	0.177	1.78 (0.77 to 4.15)	0.178
Intervention arm					
Control	29/117 (24.8%)				
Pleural vent	34/114 (29.8%)	1.29 (0.72 to 2.3)	0.391	1.54 (0.79 to 3.01)	0.209
Previous pneumothorax					
No	44/172 (25.6%)				
Yes	19/58 (32.8%)	1.42 (0.74 to 2.71)	0.290	1.7 (0.8 to 3.61)	0.165
Size of initial pneumothorax					
<4 cm	28/96 (29.2%)				
≥4 cm	35/135 (25.9%)	0.85 (0.47 to 1.53)	0.586	0.85 (0.42 to 1.74)	0.660
Long symptom duration					
No (‘same day’)	32/96 (33.3%)				
Yes (>1 day)	31/135 (23%)	0.60 (0.33 to 1.07)	0.083	0.39 (0.18 to 0.81)	0.012
Smoking status					
Never	25/72 (34.7%)				
Current or ex-smoker	37/156 (23.7%)	0.58 (0.32 to 1.08)	0.084	0.58 (0.28 to 1.19)	0.135

Variables (continuous)	Mean (SD)	Odds ratio per 10 unit increase (95% CI)*	P value	Odds ratio per 10 unit increase (95% CI)*	P value
Duration of symptoms before presentation (days)					
Resolved N=167	2.5 (5.1)				
Failed N=63	3.5 (10.1)	1.02 (0.98 to 1.06)	0.311	1.01 (0.96 to 1.06)	0.645
Baseline breathlessness (0–100)					
Resolved N=152	43.3 (27.8)				
Failed N=61	38.9 (28.8)	0.95 (0.85 to 1.05)	0.303	0.91 (0.78 to 1.06)	0.219
Baseline pain (0–100)					
Resolved N=152	39 (29)				
Failed N=61	42.1 (28.1)	1.00 (0.90 to 1.10)	0.950	1.05 (0.91 to 1.2)	0.513
Heart rate (beats per minute)					
Resolved N=161	84.1 (15.6)				
Failed N=60	78.4 (15.5)	0.78 (0.63 to 0.96)	0.017	0.79 (0.63 to 1.00)	0.047
Respiratory rate (breaths per minute)					
Resolved N=161	19.5 (4.5)				
Failed N=60	82.6 (15.7)	0.62 (0.29 to 1.31)	0.210	0.85 (0.37 to 1.92)	0.688
Systolic blood pressure (mm Hg)					
Resolved N=161	129.3 (15.9)				
Failed N=59	125.3 (14.4)	0.85 (0.69 to 1.03)	0.095	0.9 (0.72 to 1.12)	0.342

* Except for duration of symptoms which is per unit.

### Duration of treatment

The median treatment duration was 3 days (IQR 1–6). Same-day patients had a longer median duration of treatment (3.5 days, 1–7) versus 2 days (0–5) for those with long symptom duration (p 0.003), in keeping with the definition of treatment failure (ongoing pneumothorax at day 4). Patients with a large pneumothorax on initial CXR (>4 cm) had longer treatment duration (3 days (1–7)) than those with moderate size pneumothorax (1 day (0–5)) (p 0.001, Mann-Whitney).

## Discussion

Our prospectively collected RCT data show that patients who presented with PSP on the same day that their symptoms began had a higher risk of treatment failure. These same-day patients tended to have higher baseline breathlessness and pain scores but the severity of the symptoms was not independently associated with the risk of treatment failure. It should be noted that in the small group of patients (n=17) with symptoms for >7 days prior to admission also had a higher risk of treatment failure but this was not statistically significant.

### Size of pneumothorax

Large pneumothorax size was associated with increased risk of failure of needle aspiration in two small retrospective case series.^8 9^ A larger retrospective of 253 patients with PSP found higher failure rates in those with massive (>62.5%) compared with small pneumothoraces.^10^ In our study, although patients with larger pneumothorax had a longer median treatment duration, there was no increased risk of treatment failure.

### Symptom scores

There are no previous studies assessing the association of symptom severity and risk of treatment failure. Our data were prospectively collected as part of an RCT, with good completion rates (>90%). While same-day patients had higher symptom scores than those presenting later, we did not find an association between the VAS-measured symptom severity scores and treatment failure on multivariable analysis.

### Vital signs

There were no significant differences in respiratory rate or systolic blood pressure. There was a statistically significant association between *lower* heart rate and treatment failure. The effect size was small and unlikely to be clinically significant: one might expect patients who were more symptomatic to have a higher heart rate, so difficult to rationalise clinically.

### Prolonging the air leak?

The biological mechanisms resulting in same-day patients having a higher risk of treatment failure are not clear. One possible mechanism is that by inserting a device to drain the pneumothorax, a more negative pleural pressure is created, resulting in impaired healing of the pleural defect and persistent air leak.^11^

### Time to change practice?

What is different about patients who present on the same day as their symptom started? It could be that by intervening ‘early’ that we are prolonging the air leak (as above). More anxious patients may be less likely to adopt a ‘wait and see’ approach. We do not routinely record the evolution of the patient’s symptoms – that is, are they increasing with time or fluctuating. Therefore, there may be other patient factors which are not being captured on our VAS symptom scores, which are influencing the patients’ decisions to attend as soon as symptoms start. Should we delay treatment in patients with PSP? One RCT of needle aspiration versus chest drain had a ‘delayed treatment’ arm of 2–3 days; however, no difference in immediate success rates was observed.^12^

What about those patients in our study whose symptoms began >7 days ago? In theory, the majority of those patients should have had sufficient time for the air leak to heal. Patients in this situation in our study had an increased rate of ongoing air leak (treatment failure), albeit not statistically significant in this small patient group.

The ‘holy grail’ for pneumothorax management is a non-invasive way to identify those patients with ongoing air leak but this is an area of ongoing research.

The strengths of this study are the prospective nature of clinical data collection (including the first-ever daily symptom scores) from the largest RCT of PSP initial management in Europe and good data completeness. Definitions of treatment failure were predetermined and consistent across all recruiting centres. The limitations include the generalisability of the data as only patients enrolled into the RCT were included, although patient demographics are equivalent to previous epidemiological studies. Although the 100 mm VAS scores for pain and breathlessness are widely used in pleural disease, they have not been specifically validated in pneumothorax, so the MCID has not been determined. Despite 236 patients being included, only 60 patients experienced treatment failure. Therefore, there is a risk of type I error rate inflation (ie, a false-positive result) by multiple comparisons and type II errors (false rejecting other significant variables by relatively small sample size) in this analysis. The results need to be validated in another larger high-quality dataset.

## Conclusion

Patients with PSP were more likely to fail treatment if they presented on the same day that their symptoms started. Delayed treatment may allow the air leak in PSP to heal itself and thereby reduce the risk of prolonged air leak and failure of medical management.

## Supplementary material

10.1136/bmjresp-2024-003089 online supplemental file 1

## Data Availability

Data are available upon reasonable request to the corresponding author.
